# On the Necessity of Atmospheric Air for the Evolution of the Chick in the Incubated Egg

**Published:** 1840-07

**Authors:** 


### Art. XIV.

*De Necessitate Aëris Atmosphericæ ad Evolutionem Pulli in Ovo incubito. Dissertatio Inauguralis Physiologica.* Auctore Theod. Schwann.—*Berolini,* 1834. 4to, pp. 32.

*On the Necessity of Atmospheric Air for the Evolution of the Chick in the Incubated Egg.* By Th. Schwann.—*Berlin.*

We are induced to present our readers with a brief notice of this Thesis, although it is not now of very recent date, in consequence of the rather startling result of the experiments lately performed by Mr. Towne, (Guy's Hospital Reports, Oct. 1839; and Br. and For. Med. Review, Vol. IX. p. 396.) It is maintained by that gentleman that the access of oxygen to the contents of the egg, through the pores of the shell, is not, as commonly believed, necessary to its development; since the shell may be enveloped in coatings which would seem impervious to gas, without the slightest interruption or retardation of the usual processes. Moreover he affirms that the *folliculus aëris* within the shell remains unbroken until the nineteenth day, when it supplies the chick with the means of filling its lungs with air. This last proposition we think reasonable; it is the former one to which we take exception.

Mr. Towne's experiments were made by laying upon the shell several coats of albumen rendered consistent by evaporation; and over these were placed several layers of paper soaked in albumen until they were sufficiently soft to be applied closely to the convex surface. "The whole," he says, "formed a covering so thick and horny, that I felt convinced it was entirely impermeable." On this point we take leave to differ from Mr. Towne. Experience has shown that many substances which appear entirely destitute of pores are susceptible of permeation by gases; especially when gases of different kinds are in contact with their opposite sides. A solid plug of plaster of Paris, three quarters of an inch thick, is traversed by hydrogen with a rapidity truly wonderful, if oxygen or atmospheric air be on the other side of it; and we can readily conceive that even Mr. Towne's horny envelope was susceptible of slow permeation by oxygen, when carbonic acid had to be disengaged from the egg. The experiment proves nothing, until it is demonstrated that the surrounding air undergoes no change. Until this is shown we consider ourselves quite at liberty to affirm that the envelope *is* permeable; since all unexceptionable experiments concur in showing that the evolution of the chick is prevented by any means which *certainly* prevents the passage of atmospheric air through the shell. We do not think the coatings of oil-paint, placed by Mr. Towne in some of his experiments outside the layers of albuminized paper, a more perfect security than the first envelope. The paint was composed of white lead, with a large proportion of sugar of lead used as a drier. "This," says Mr. Towne, "I did with the double intention of offering an additional obstruction to the air, and also to prove whether the paper was or was not entirely sufficient for this purpose; concluding that if it were not the fume from so noxious an application must inevitably prevent the progress of incubation." Now on the first point we remark that the rapid drying of the coats of paint renders it to our minds extremely probable that such a number of minute cracks would be formed in each layer, as to render the whole pervious to air; and on the second we would observe that no such noxious fumes as Mr. Towne supposes would be exhaled from his composition. The matter will be very easily tested. Let Mr. Towne experiment upon one of Professor Graham's diffusion-tubes instead of upon eggs; let him tie his layers of paper over the extremity, and put his coats of oil-paint over these, and then fill the tube with carbonic acid gas; and we venture to affirm that, after some days, he will find that the contents of the tube have been changed by the exosmose and endosmose of gases through its closed extremity.

A much more perfect means of excluding the air from the interior of an egg is that which is commonly practised in this country—the rubbing of the shell with grease. This is done for two purposes: either to prevent decomposition, when it is desired to preserve the eggs for some time, or to prevent chickens from being reared from them. As to its success in both these objects we can bear personal testimony. In regard to the first, the effect will be more complete the sooner the application is made after the egg has been laid. We have eaten eggs, thus prepared in England, off Barbadoes, after being at sea for a month, which were certainly superior to fresh eggs brought off from the island. Moreover it is a common practice with farmers' wives in the country to employ the same application as a means of preventing those who purchase their eggs from rearing from them some peculiar breed of poultry valued by the seller; and we have been assured by competent authority that this plan is never known to fail. Considering the quantity of oily matter already contained in the egg, we cannot imagine that the application of simple grease can exert any noxious effect upon the embryo; more especially since its power of preventing decomposition is so remarkable.

Evidence yet more satisfactory, however, is derived from experiments upon the artificial incubation of eggs in irrespirable but innocuous gases. Hydrogen and nitrogen are the only ones which can be regarded in this light; carbonic acid has a manifestly deleterious effect upon the embryo as upon the adult. Among all the experiments of this nature which have yet been made, we believe those of Schwann to be the most unexceptionable, both in regard to the plan on which they were devised, and the care with which they were conducted. The eggs were placed in a capacious glass vessel, to which a brass top was accurately fitted. The non-permeability of this vessel had been tested by leaving it for three days exhausted of air. The brass cover was fitted with two stopcocks: one of which communicated with a reservoir of gas, the purity of which was carefully ascertained, and the other with an air-pump. The air was then exhausted; and thus not only the atmosphere surrounding the egg was removed, but the air contained within it. That this process was not in itself deleterious, however, was shown by the fact that eggs submitted to it were capable of proceeding in their subsequent development, if again exposed to atmospheric air. Into the exhausted receiver nitrogen or hydrogen gas was then admitted; and this was again drawn out by the air-pump. This process was repeated five or six times; so that the complete displacement of the oxygen was secured. The glass vessel was then placed in an apparatus adapted to maintain its temperature at the requisite height without intermission. The following is the result of numerous experiments:

No eggs went through their full development; but in the greater number the primary changes had occurred; so that the presence of oxygen seems necessary, not so much to give the stimulus to the development as to maintain it. It appeared that the separation of the germinal membrane into the serous and mucous layers, and the formation of the *area pellucida* might thus take place; but in no instance was the formation of the vascular layer perceived. The point at which they stopped is that which eggs incubated in atmospheric air reach about the fifteenth hour. But it would appear that eggs incubated in hydrogen do not lose their vitality at that period; for, if removed at the twenty-fourth hour into atmospheric air, their development may continue; though this will not occur if the change be delayed until about the thirtieth hour. It would seem, therefore, as if the development is retarded during its continuance, as well as finally checked, by the want of oxygen. The liberation of a small quantity of carbonic acid was observed in all the experiments; and this was found to take place from the very beginning of the process; and not to be a product of decomposition.

Further experiments on this subject are much wanting, to determine the changes which are really produced in the surrounding atmosphere during incubation in gases containing oxygen; and we trust that this deficiency will not long remain unsupplied. We call Mr. Towne's attention to this point.

---

